# Association of GCLM -588C/T and GCLC -129T/C Promoter Polymorphisms of Genes Coding the Subunits of Glutamate Cysteine Ligase with Ischemic Heart Disease Development in Kazakhstan Population

**DOI:** 10.1155/2017/4209257

**Published:** 2017-07-05

**Authors:** Liliya Skvortsova, Anastasia Perfelyeva, Elmira Khussainova, Alma Mansharipova, Henry Jay Forman, Leyla Djansugurova

**Affiliations:** ^1^Institute of General Genetics and Cytology, Al-Farabi St. 93, Almaty, Kazakhstan; ^2^Russian Medical University, Torekulovst 71, Almaty, Kazakhstan; ^3^Leonard Davis School of Gerontology, University of Southern California, 3715 McClintock Avenue, Los Angeles, CA 90089, USA

## Abstract

**Background:**

Glutamate cysteine ligase (GCL) is a rate-limiting enzyme in synthesis of glutathione. Evidence suggests that genetic variations in the promoter region of genes coding a catalytic subunit (GCLC -129T/C) and a modifier subunit (GCLM -588C/T) of GCL have a functional impact on their transcriptional activity and were associated with various disorders. Hence, we hypothesize whether these two polymorphic variants of GCLM and GCLC genes are associated with the risk of ischemic heart disease (IHD) development in the population of Kazakhstan.

**Methods:**

We evaluated 360 patients with IHD and 341 control subjects. Allele frequencies of studied promoters' polymorphisms were detected by PCR-RFLP analysis. Multiple logistic regression analysis was applied to assess the risk for different genotypes obtained.

**Results:**

The presence of -588T allele in GCLM and -129T allele in GCLC gene genotypes was associated with an increased risk of IHD (GCLM -588T: OR = 3.92, *p* = 0.003; GCLC -129T: OR = 3.22, *p* = 0.03) for general ethnically mixed group. Analysis of each ethnical groups separately showed the higher risk tendency for Kazakhs as for GCLM -588T (OR = 4.79; *p* = 0.03) and as for GCLC -129T (OR = 4.79, *p* = 0.03). For Russians, statistically differences for two polymorphisms were not observed.

**Conclusion:**

The two promoter polymorphisms of GCLM (-588C/T) and GCLC (-128T/C) are associated with an increased risk of IHD in Kazakhstan population.

## 1. Introduction

Nowadays, ischemic heart disease (IHD) is a major health problem due to the high prevalence of morbidity and mortality both in economically developed countries and in Kazakhstan. According to the Statistics Agency of Kazakhstan, in 2016, the death rate from IHD was 179.30 per 100 thousand people [1]. Despite the fact that the opening of the classic risk factors has reduced mortality from IHD in many developed and developing countries, the incidence of IHD continues to grow steadily every year. Perhaps, this is due to that IHD is a multifactorial disease and the body's response to these environmental risk factors is under individual genetic control. Genetic variations are of particular interest because they provide insights into personalized approach to prevention and treatment of IHD. Therefore, one of the important areas in this field is an active search for new genetic risk factors predisposing to IHD development.

Growing evidence shows that failure of antioxidant defense mechanisms may determine the pathogenesis of virtually every stage of IHD formation [2–4]. In our study, we turned our attention to the largest reservoir of reducing equivalents in cells—glutathione (GSH).

GSH is the principal substrate for the evolutionarily ancient antioxidative system in the body. GSH is composed by three amino acids (L-cysteine, L-glutamic acid, and glycine) and exists in most mammalian cells in two major forms, reduced GSH and the disulfide (oxidized) GSSG [5]. Biosynthesis of the GSH takes place in cells via of two-step ATP-dependent reactions catalyzed by glutamate cysteine ligase (GCL) and glutathione synthetase (GSS). Bioavailability of the first enzyme is essential for de novo GSH synthesis as it is a rate-limiting enzyme. GCL is a heterodimer made up of two different subunits, namely, a catalytic subunit (GCLC) and a kinetic modulating subunit (GCLM) [6]. Enzymatic availability of these subunits is influenced by expression on transcription level in redox sensitive manner via putative oxidative stress-responsive elements in their promoter/enhancer regions (antioxidant/electrophile responsive elements (ARE/EpRE)) [7, 8]. The importance of the promoters is clear for gene expression, and the presence of SNP in these regions may have an influence on the synthesis of mRNA and its bioavailability [9]. Several SNPs for GCLC and GCLM promoters have been identified that have possible associations with cardiovascular disease development in case-control studies. Two polymorphisms in GCLM gene promoter -588C/T and in GCLC gene promoter -129C/T were revealed in 2002 and 2003 by Nakamura et al. [10] and Koide et al. [11], respectively. Later, the functional significance of these polymorphisms was confirmed by experimental [12] and case-control studies [13–15]. However, many aspects of the relationship of this kind of polymorphisms with the development of cardiovascular pathologies, as well as its prevalence in the different populations, are contradictory and require further studies. Thus, the aim of the present study was to evaluate the significance of GCLC -129C/T and GCLM -588C/T promoter polymorphisms in IHD development in Kazakhstan population.

## 2. Materials and Methods

### 2.1. Study Subjects

This study was done on the basis of “City Clinical Hospital Number 1” (Almaty, Kazakhstan), cardiology department, and “Kazakh-Russian Medical University.” Peripheral blood samples were collected from 360 patients with stable forms of angina pectoris (angina pectoris of I–IY classes according to the Canadian classification of cardiologists and Association of Cardiologists of the Republic of Kazakhstan [16]), with chronic heart failure of II-III classes [17] and postinfarction cardiosclerosis. Diagnosis of IHD was based on WHO criteria. All of the patients underwent diagnostic stress tests or coronary angiography, evaluation of the presence of a chest pain syndrome, confirmed by ECG data, and cardio-specific enzymes (troponin, myoglobin, and creatine kinase). Exclusion criteria included the presence of concomitant pathology as diabetes, inflammatory diseases, local inflammatory processes, allergic reactions, signs of renal insufficiency, circulatory insufficiency of stage III, Alzheimer's disease, presence of hyperthermia, and insolations during the previous 2-3 weeks.

Control blood samples were obtained from 341 healthy donors without clinical manifestations of IHD and without family history of atherosclerosis and ischemic events at ECG, oncology, autoimmune diseases, any hereditary diseases, and acute/chronic inflammatory diseases. Control group was selected to the patient group according to personal data obtained from questionnaires.

Detailed questionnaires used contained basic information including ethnicity, demographic status, habits (tobacco, alcohol, and diet), and other illness. Collection of biomaterials was performed on a voluntary informed consent to participate in the research. This research work was approved by the ethics committee (Kazakh-Russian Medical University local ethics committee, Almaty (Protocol number 36 of January 5, 2016)).

### 2.2. Genomic DNA Isolation

DNA was isolated from EDTA-treated peripheral blood samples by using Genomic DNA Purification Kit (Fermentas). Qualitative and quantitative characteristics of the DNA samples were estimated by spectrophotometry (Eppendorf Biophotometer plus). Isolated DNA samples were stored at −20°C.

### 2.3. Genotyping

Genotypes of each promoter SNP polymorphism were determined by PCR-RFLP method. 50 ng of target genomic DNA was amplified in 20 *μ*l PCR mixture, containing 10 *μ*l 2× PCR Master Mix (0.05 U/*μ*l Taq DNA polymerase reaction buffer, 4 mM MgCl2, and 0.4 mM of each dNTP (Thermo Scientific)) and 5 pM of each specific primer. For the amplification of regions capturing the GCLM (-588C/T) and GCLC (-129T/C) polymorphic sites, we used primers as described by Nakamura et al. [10] and Koide et al. [11], respectively. The following optimal conditions were selected for the PCR reaction for both genes' polymorphisms: initial denaturation 3 min at 95°C, with the following 35 cycles of denaturation 1 min at 95°C, annealing for 30 sec at 58°C (GCLM) or 61°C (GCLC), and extension for 1 min at 72°C. In the second stage, for the restriction of the PCR products, endonuclease *MspI* was used for GCLM -588C/T site and *Tsp45I* for GCLC -129T/C site, according to the manufacturer's recommendations. Analysis of restriction fragments was performed in 8% polyacrylamide (Sigma-Aldrich, USA) gel electrophoresis with subsequent visualization under UV light. Allelic variants of the genes were identified by genotype-specific restriction fragments: for GCLM genotype CC—200, 84, and 45 bp; CT genotype—200, 129, and 84 bp; genotype TT—200 and 129 bp. Genotype-specific fragments for GCLC: CC genotype—500 and 113 bp; CT genotype—500, 302, 198, and 113 bp; TT genotype—302, 198, and 113 bp.

Genotyping of all samples was performed in duplicate and scored by different individuals to avoid genotyping error.

### 2.4. Statistical Analysis

Values characterized by gender, ethnicity, and smoking habits of the case and control populations were calculated in percentage, and the rest were expressed as mean ± standard deviation or standard error values. The Student *t*-test was used to compare the distribution of variables between case and control cohorts. A *p* value of less than 0.05 was considered as significant.

The expected genotype frequencies for case and control cohorts were calculated in accordance with standard Hardy-Weinberg equilibrium using the conventional Pearson's chi-square test (*χ*2).

Estimation of the coefficient of relative risk was calculated by the method of “odds ratio” (OR) in conjunction with an estimate 95% confidence interval (95% CI) and the “chi-square” test (*χ*2) for the degrees of freedom = 1. The ORs for an individual genotypes and allele frequencies were calculated. We performed multivariate analysis using the general model (analysis of each allele, genotype separately), the dominant model (normal homozygotes versus combination of heterozygotes with polymorphic homozygotes), and the recessive model (combination of normal homozygotes with heterozygotes versus polymorphic homozygotes). Separate analyses were done for main ethnic groups (Kazakh and Russian). A *p* value of <0.05 was considered statistically significant.

## 3. Results

### 3.1. Cohorts' Characteristics

A total of 360 IHD patients with angina pectoris and 341healthy subjects were selected for the “case-control” study. The characteristic of IHD case and control groups are reported in Table 1.

All of the patients and control cohorts were divided into three main ethnic groups: Kazaks, Russian, and other Asians (Koreans, Tatars, Uighurs, and Kyrgyz). Ethnic's ratios in both groups were almost identical: 63.88% Kazakhs, 26.94% Russians, and 8.21% other Asians in IHD cohort and 69.5% Kazakhs, 19.94% Russians, and 9.68% other Asians in control cohort.

### 3.2. GCLM (-588C/T) and GCLC (-129T/C) Genotyping Results

Genotyping of GCLM (-588C/T) and GCLC (-129T/C) genes' promoter polymorphisms showed that the distribution of genotype frequencies corresponds to Hardy-Weinberg equilibrium, both among the controls (GCLM (-588C/T): *χ*2 = 1.397, *p* = 0.497; GCLC (-129T/C): *χ*2 = 0.456, *p* = 0.796) and the experimental group (GCLM (-588C/T): *χ*2 = 0.531, *p* = 0.725; GCLC (-129T/C): *χ*2 = 0.204, *p* = 0.701).

An associative analysis of the data was carried out using multiplicative, general, dominant, and recessive models.

According to the multiplicative model of inheritance, the risk (OR) is expressed equally for T allele in both genes in the total mixed group: for GCLM -588T allele—OR = 1.53 (95% CI = 1.15–2.03, *χ*2 = 8.743, *p* = 0.003); for GCLC -129T allele—OR = 1.48 (95% CI = 1.09–2.03, *χ*2 = 6.212, *p* = 0.013). Whereas, C allele in both polymorphisms demonstrates a protective effect: GCLM -588C allele—OR = 0.65 (95% CI = 0.49–0.87, *χ*2 = 8.743, *p* = 0.003) and GCLC -129C allele—OR = 0.67 (95% CI = 0.49–0.92, *χ*2 = 6.219, *p* = 0.013).

Table 2 shows the association results for general ethnically mixed IHD patients and control groups (*χ*2 test, df = 2). General inheritance model revealed an increasing negative impact of the T allele for each genotype in both gene polymorphisms: for GCLM gene in the presence of one T allele in heterozygote (CT), OR was slightly high—1.44 (*p* = 0.009), and in homozygote state (TT), it was already twice increased (OR = 2.91, *p* = 0.009); for GCLC gene in CT genotype risk, OR was 1.35 (*p* = 0.03), whereas in TT genotype, it was two and a half times higher (OR = 3.22, *p* = 0.03).

Combination of genotypes CC + CT confirmed these findings and showed a really strong protective (positive) effect for C allele for both genes GCLM (OR = 0.34, *p* = 0.05) and GCLC (OR = 0.31, *p* = 0.06). This was completed by the receive model where CT + TT combination has reduced risk properties for GCLM (OR = 1.57, *p* = 0.006) and GCLC (OR = 1.47, *p* = 0.03) genes, because of the positive impact of C allele in CT genotype.

As the investigated case-control groups are heterogeneous in ethnicity, age, gender, and smoking status, we did a separate association analysis for both genes' polymorphisms for Kazakh and Russian cohorts (Table 3), for two age groups (younger than 45 and older than 45), and for males/females and smokers/nonsmokers presented in Tables S1 and S2 available online at https://doi.org/10.1155/2017/4209257. Association analysis for both genes' polymorphisms for Kazakh and Russian ethnicity based on the dominant and recessive inheritance models is also presented in Tables S1 and S2.

According to the general model, GCLM -588TT genotype is associated with high risk of IHD in Kazakh subgroup (OR = 4.23) and GCLM -588CC genotype shows a strong protective effect (OR = 0.67), which is strengthened by dominant model combination CC versus CT + TT (OR = 0.24, 95% CI = 0.05–1.12, *χ*2 = 3.866, *p* = 0.05). In Kazakh, the presence of at least one T allele in genotype increases the risk of IHD development. Russian showed a tendency to the elevation of the IHD risk association (OR = 1.41) especially in the recessive model (OR = 1.46, 95% CI = 0.77–2.76, *χ*2 = 1.376, *p* = 0.2). CC genotype also had protective properties as in the general model (OR = 0.68) and as in the receive model (OR = 0.68), but all these data obtained for Russian are not statistically significant (*p* > 0.5).

Also, we found that GCLM -588TT genotype was associated statistically with higher risk of IHD in females (OR = 3.97, 95%CI = 0.85–18.54, *χ*2 = 10.812, *p* = 0.004) and in individuals older than 45 years (OR = 2.91, 95%CI = 0.80–10.53, *χ*2 = 6.097, *p* = 0.04). Analysis of individuals' smoking status did not reveal any association with IHD development (Table S1).

For the GCLC -129TC promoter polymorphism, a negative risk impact is expressed for -129T allele (OR = 1.62, 95% CI = 1.10–2.40, *χ*2 = 5.917, *p* = 0.015) and -129TT genotype was strongly associated with the high risk in Kazakhs by the general model (OR = 4.79, 95% CI = 1.02–22.39, *χ*2 = 6.660, *p* = 0.04), confirmed by the dominant model (OR = 4.79, 95% CI = 1.02–22.39, *χ*2 = 4.781, *p* = 0.03). 129C allele carriers demonstrated a clear protective effect according to the multiplicative model (OR = 0.62, 95% CI = 0.42–0.91, *χ*2 = 5.917, *p* = 0.015), and the presence of this allele reduces the negative impact of -129T allele especially for combination CC + CT versus TT (OR = 0.21, 95% CI = 0.04–0.98, *χ*2 = 4.781, *p* = 0.029).

For Russians, ethnical subgroup genotypes did not show any associations in different models.

Also, the data detected a significant association between the group older than 45 years and -129TT genotype in the general model (OR = 7.15, 95% CI = 0.90–56.80, *χ*2 = 5.597, *p* = 0.06) confirmed by the receive model (OR = 7.15, 95% CI = 1.02–22.39, *χ*2 = 4.694, *p* = 0.03) (Table S2). In women, the data showed a positive correlation for -129T allele with IHD development according to the multiplicative model (OR = 1.63, 95% CI = 1.12–2.37, *χ*2 = 6.566, *p* = 0.01), whereas -129C allele had a protective effect (OR = 0.61, 95% CI = 0.42–0.89, *χ*2 = 6.566, *p* = 0.01). IHD risk was expressed for -129TT genotype (OR = 2.33, 95% CI = 0.61–8.89, *χ*2 = 6.376, *p* = 0.04).

There were no IHD risk associations of GCLC -129T/C genotypes with tobacco smoking status (Table S2).

## 4. Discussion

Growing evidence shows that imbalance in production of free radicals and expression dynamics of antioxidant enzymes may determine the pathogenesis of virtually every stage of IHD formation [2–4]. GSH seems to be an important in reducing free radical overproduction and extensively studied worldwide [18]. Critical point in GSH biosynthesis is the availability of the GCL enzyme, as a key enzyme in its synthesis [5]. It was reported that SNPs in the promoters 5′-flanking region of GCLC (-129T/C) and GCLM (-588C/T) genes, coding the subunits of GCL, demonstrated specific correlations with the impaired gene promoter activity [10, 11] and with the development of many pathological disorders revealed by associative studies [19–21].

We tried to clarify whether the GCLC (-129T/C) and GCLM (-588C/T) polymorphisms are involved in IHD predisposition, through the imbalance of GSH antioxidant pathways, in population from Kazakhstan by associative study.

In case-control epidemiological studies, selection of cohorts is critical for all further results. These groups should be matched to each other in many parameters in order to assure more reliable associations with the disease development and to avoid undesirable impacts from other influencing factors. In our study, only individuals from one geographic zone (Almaty City) of Kazakhstan were chosen. To create control and IHD patient cohorts, we used the main population characteristics and parameters that were thoroughly matched to each other for the minimization of ethnicity, age, sex, tobacco smoking effects, and other states that may cause an oxidative stress and IHD. Such important for IHD susceptibility, physiological parameters such as total cholesterol level and blood pressure were statistically different between the two groups. Unfortunately, the more detailed data on lipoprotein panel of blood (LDL, HDL, and triglycerides) were not available for us, because the data were not provided by physicians.

Case-control genotyping provides information on the distribution of allele frequencies in a population of healthy individuals. We compared revealed allele frequencies of the genes with data from literature and NCBI SNP database (Table 4). -588C allele frequency for GCLM -588C/T polymorphism is closer to frequencies of known Asian populations (0.836–0.906) than of Europeans; the frequency of -588T allele is between the frequencies of known Asian and European populations. For GCLC -129T/C polymorphism, both alleles -129C and -129T frequencies are different from Asian and European populations: -129C allele frequency is lower than that of Asians and Europeans whereas -129T is higher. These differences may be caused by ethnicity heterogeneity of our population, by different ethnographic, evolutionary peculiarities, and lifestyle background.

According to the associative analysis, GCLC -129TT and GCLM -588TT genotypes demonstrated high-positive associations with IHD development in general ethnically mixed population. Wherein, the presence of one T allele in genotype increases the risk: for GCLC gene in CT genotype risk is 1.35 (95%CI = 0.95–1.92, *χ*2 = 6.735, *p* = 0.03), whereas in TT genotype, it is two and a half times higher (OR = 3.22, 95%CI = 0.88–11.80, *χ*2 = 6.735, *p* = 0.03). For GCLM gene in the presence of one T allele in heterozygote (CT), OR is slightly high—1.44 (*p* = 0.009), and in homozygote state (TT), it is already twice increased (OR = 2.91, *p* = 0.009). These findings are consistent with the most associated data obtained previously in different pathological states.

As mentioned before, two pioneering studies done by Koide et al. [11] and Nakamura et al. [10] established lower promoter activities in cells for GCLC -129T allele carriers and for GCLM -588T allele carriers. This observation was supported by Butticaz et al. for GCLC -129C/T polymorphism [12]. In further studies, they established the similar negative effect of -588T polymorphism on endothelium-dependent vasomotor reactivity in large and resistance coronary arteries (abnormally low dilation effect or high constriction) and may affect to coronary artery disease development [22]. Previous studies suggested the association of these polymorphisms with the development of various diseases. The -129T allele variant in GCLC gene may cause susceptibility to MI [11], renal disease in patients with type 1 diabetes mellitus [23], schizophrenia [20], cystic fibrosis [24], chronic obstructive pulmonary disease [19], and nonalcoholic steatohepatitis in Brazilian patients [21]. The GCLM -588C/T promoter polymorphism has been observed in associations with asthma [14], MI in type 2 diabetic patients together with other prooxidant gene polymorphisms [15], and nonfamilial idiopathic dilated cardiomyopathy [25, 26]. In a case-control study, done by Engström et al., low effect of GCLM -588C/T polymorphism on MI risk was found only in combination with EPA + DHA acid plasma levels and erythrocyte-methylmercury levels [27]. Muehlhause et al. [28] did not find association of GCLM -588C/T polymorphism with the risk and extent of IHD in a German cohort. Mansego et al. did not reveal associations of GCLM -588C/T polymorphism with blood pressure values and hypertension in a population-based study including 1388 participants [29]. Thus, the association apparently depends on additional unknown factors that vary among populations.

Currently, the question remains unclear about the ethnic influence on association studies. According to the available case-control studies on cardiovascular diseases, high-association results were obtained for GCLM -588TT genotype with cardiomyopathy in the Japanese population (OR = 3.13) [25] and for GCLC -129TT with MI also in the Japanese population OR = 1.81 [11]. In our study, detailed analysis of dominant ethnic groups revealed that high ORs were detected for Kazakhs (GCLC -129TT: OR = 4.79, *p* = 0.04; GCLM -588TT: OR = 4.23, *p* = 0.04), but not for Russians (GCLC -129TT: OR = 0.69, *p* = 0.8; GCLM -588TT: OR = 1.41, *p* = 0.5). Interpreting these results should also take into account the specificity of nutrition aspects of Kazakhs: traditionally, food of this ethnic group is still enriched with fatty meat and low vegetable consumption. Also, this may be explained by the limited and small number of the Russian group in the mixed population (case (*n* = 102), control (n =71)) suggesting the benefit of further studies of a large case-control group. Different ethnic heterogeneity associations make it necessary to do additional case-control studies on larger groups of each ethnic cohort separately.

The precise mechanisms by which -129T allele of GCLC and -588T allele of GCLM gene might decrease *gclc* and *gclm* subunit expression are still unclear. Although the GCLC and GCLM gene promoters have identified several antioxidant/electrophile responsive elements (ARE/EpRE), AP-1, AP-2 elements [30]. These nucleotide substitutions may alter the interactions among the *cis* elements and their stress-responsive transcription factors and modulators Nrf2 [30, 31], Maf G/F/K and c-Jun [31, 32], c-Myc [33], and AP-1/2 [34]. As a result, reduced *gclc* and/or *gclm* subunit expression leads to the inconsistent assembly of GCL, the main regulator of GSH synthesis. This becomes important in a constant exposure to many stress factors where inadequate biosynthesis of GSH and its reduced quantity may provoke different pathological states in the body. In addition, features of human genetic and physiological variations may determine different significance of GSH system in normal conditions and pathology.

The data in the current investigation, along with the previous studies, strongly support this hypothesis and show the importance of GSH antioxidant pathways in IHD pathology. Perhaps, the ethnic peculiarities of lifestyle and diet should be taken in account regarding the GCL genotypes susceptibility to IHD diseases. It should be also considered that other nucleotide changes identified in the promoter regions of GCLC and GCLM genes may have an impact on expression and thus on IHD associative studies [35]. Moreover, besides commonly used parameters for cardiovascular diseases such as main symptoms, blood pressure, ethnicity, age, gender, and smoking status, it is necessary to obtain and consider detailed information on lipoprotein panel, fasting glucose, angiography, medical taking, and diet, so as to obtain as much information as possible on all factors that may influence antioxidant homeostasis.

## 5. Conclusions

Obtained data supplement the earlier studies on important functional role of GCL enzyme, depending on expression of GCLC and GCLM genes, in cardiovascular disease development. The present data demonstrate that two SNPs in the promoter regions of two subunits GCLC -129C/T and GCLM -588C/T building the active GCL enzyme are associated with IHD development in Kazakhstan ethnically mixed population with higher manifestation in Kazakh ethnic group than in Russian.

## Supplementary Material

Supplementary Tables. Table S1 Association of ethnicity, age, gender and tobacco smoking status with GCLM -588C/T (rs17883901) gene polymorphism and IHD. Table S2 Association of ethnicity, age, gender and tobacco smoking status with GCLC-129 C/T (rs41303970) gene promoter polymorphism and IHD.

## Figures and Tables

**Table 1:** Comparison of IHD patient group and control subjects.

Characteristics		IHD group	Control group	*t*_st_	*p* value
*n*		360	341		
Age (y)		1923–1970 (60 ± 12.04)	1921–1970 (52 ± 13.23)	0.51	0.699
Gender, *n* (%)	Male	91 (25.27)	109 (31.96)	1.628	0.350
	Female	269(74.72)	232 (68.03)	1.042	0.486
Ethnicity, *n* (%)	Kazakhs	230 (63.88)	237 (69.5)	0.908	0.530
	Other Asians	28 (8.21)	33 (9.68)	0.840	0.555
	Russians	102 (28.32)	71 (20.82)	1.925	0.305
Smoking status, *n* (%)	Smokers	41(11.39)	57 (16.71)	1.796	0.323
	Nonsmokers	319 (88.61)	284 (83.28)	0.758	0.587
Total cholesterol (mmol/l)		>5.02 (5.47 ± 0.09)	<5.02 (4.01 ± 0.06)	13.498	0.047
Blood pressure (mmHg)		>140/90	120/60	—	—

There were no statistically significant differences in age, gender, and smoking status between the control and experimental groups (*p* > 0.05 for all characteristics).

**Table 2:** Odds ratios (ORs) and 95% confidence intervals (CIs) for GCLM (-588C/T) and GCLC (-129T/C) genes in studied general mixed groups.

Gene polymorphism	Genotype	Controls	IHD patients	OR (95% CI)	*p* value
GCLM (-588C/T)	CC	250	229	0.64 (0.46–0.88)	0.009
	CT	87	119	1.44 (1.04–2.00)	
	TT	4	12	**2.91** (0.93–9.10)	
GCLM (-588C/T) dominant model	CC + CT	337	348	0.34 (0.11–1.08)	0.05
	TT	4	12	2.91 (0.93–9.10)	
GCLM (-588C/T) receive model	CC	250	229	0.64 (0.46–0.88)	0.006
	CT + TT	91	131	1.57 (1.14–2.17)	
GCLC (-129T/C)	CC	268	257	0.68 (0.48–0.96)	0.03
	CT	70	93	1.35 (0.95–1.92)	
	TT	3	10	**3.22** (0.88–11.80)	
GCLC (-129T/C) dominant model	CC + CT	338	350	0.31 (0.08–1.14)	0.06
	TT	3	10	3.22 (0.88–11.80)	
GCLC (-129T/C) receive model	CC	268	257	0.68 (048–0.96)	0.03
	CT + TT	73	103	1.47 (1.04–2.08)	

**Table 3:** Odds ratios (ORs) and 95% confidence intervals (CIs) for GCLM (-588C/T) and GCLC (-129T/C) genes based on Kazakh and Russian ethnicity (general inheritance model).

Groups	Genotypes	Case/control, *n*	OR (95% CI)	*χ*2	*p* value
*GCLM -588C/T*					
Ethnicity					
Kazakh	CC	156/180	0.67 (0.44–1.00)	6.211	0.04
	CT	66/55	1.33 (0.88–2.02)		
	TT	8/2	**4.23** (0.89–20.16)		
Russian	CC	60/48	0.68 (0.36–1.29)	1.388	0.5
	CT	38/21	1.41 (0.74–2.70)		
	TT	4/2	1.41 (0.25–7.90)		
*GCLC -129T/C*					
Kazakh	CC	168/191	0.65 (0.42–1.01)	6.660	0.04
	CT	53/44	1.31 (0.84–2.06)		
	TT	9/2	**4.79** (1.02–22.39)		
Russian	CC	66/49	0.82 (0.43–1.57)	0.473	0.8
	CT	35/21	1.24 (0.65–2.39)		
	TT	1/1	0.69 (0.04–11.27)		

**Table 4:** Allele frequencies of GCLM (-588C/T) and GCLC (-129T/C) promoter polymorphism genes in Kazakhstan population (controls) and other earlier studied Asian and European populations.

Polymorphism	Alleles	Frequency of alleles		
		Control cohort (Kazakhstan population)	Data from different sources	
			Asian populations	European populations
GCLM (-588C/T)	C	0.861	0.836–0.906 [36]	0.80–0.847, [28]
	T	0.139	0.094–0.164 [36]	0.160–0.20 [28, 36]
GCLC (-129T/C)	C	0.889	0.911 [11]	0.929–0.931 [27, 37],
	T	0.111	0.089 [11]	0.069–0.071 [27, 37]
