# Supplementary material for: Association of GCLM -588C/T and GCLC -129T/C Promoter Polymorphisms of Genes Coding the Subunits of Glutamate Cysteine Ligase with Ischemic Heart Disease Development in Kazakhstan Population
Source: Dis Markers. 2017 Jul 5;2017:4209257. doi: 10.1155/2017/4209257 (PMC5516751; doi:10.1155/2017/4209257)
Supplement: Supplementary file 1 — Supplementary Tables. Table S1 Association of ethnicity, age, gender and tobacco smoking status with GCLM -588C/T (rs17883901) gene polymorphism and IHD. Table S2 Association of ethnicity, age, gender and tobacco smoking status with GCLC-129 C/T (rs41303970) gene promoter polymorphism and IHD. [file 4209257.f1.docx]

Supplementary tables

**Table S1** Association of ethnicity, age, gender and tobacco smoking status with GCLM -588C/T (rs17883901) gene polymorphism and IHD

| Groups | Inheritance model | Genotypes | Case/Control, n | | OR (95% CI) | χ2 | p-value |
| --- | --- | --- | --- | --- | --- | --- | --- |
| GCLM -588C/T | | | | | | | |
| Ethnicity | | | | | | | |
| Kazakh | Dominant | CC+CT | 222/235 | 0.24 (0.05 – 1.12) | | 3.866 | 0.04 |
|  |  | TT | 8/2 | 4.23 (0.89 – 20.16) | |  |  |
|  | Recessive | CC | 156/180 | 0.67 (0.44 – 1.00) | | 3.816 | 0.05 |
|  |  | CT+TT | 74/57 | 1.50 (1.00 – 2.25) | |  |  |
| Russian | Dominant | CC+CT | 98/69 | 0.71 (0.13 – 3.99) | | 0.153 | 0.7 |
|  |  | TT | 4/2 | 1.41 (0.74 – 2.70) | |  |  |
|  | Recessive | CC | 60/48 | 0.68 (0.36 – 1.29) | | 1.376 | 0.24 |
|  |  | CT+TT | 42/23 | 1.46 (0.77 – 2.76) | |  |  |
| Age | | | | | | | |
| ˂45 | General | CC | 21/67 | | 1.10 (0.33 – 3.70) | 0.986 | 0.6 |
|  |  | CT | 3/13 | | 0.71 (0.19 – 2.74) |  |  |
|  |  | TT | 1/1 | | 3.33(0.20 – 55.32) |  |  |
| >45 | General | CC | 208/183 | | 0.69 (0.49 – 0.97) | 6.097 | 0.04 |
|  |  | CT | 116/74 | | 1.33 (0.94 – 1.89) |  |  |
|  |  | TT | 11/3 | | 2.91(0.80 – 10.53) |  |  |
| Gender | | | | | | | |
| Males | General | CC | 62/77 | | 0.92 (0.5 – 1.69) | 0.463 | 0.793 |
|  |  | CT | 25/30 | | 1.01 (0.54 – 1.89) |  |  |
|  |  | TT | 3/2 | | 1.84(0.30 – 11.29) |  |  |
| Females | General | CC | 167/173 | | 0.55 (0.38 – 0.81) | 10.812 | 0.004 |
|  |  | CT | 94/57 | | 1.64 (1.11 – 2.42) |  |  |
|  |  | TT | 9/2 | | **3.97**(0.85 – 18.54) |  |  |
| Smoking | | | | | | | |
| Yes | Recessive | CC | 26/45 | | 0.50 (0.20 – 1.23) | 2.331 | 0.1 |
|  |  | CT+TT | 14/12 | | 2.02 (0.81 – 5.02) |  |  |
| No | General | CC | 203/205 | | 0.67 (0.47 – 0.94) | 5.758 | 0.05 |
|  |  | CT | 108/75 | | 1.42 (1.00 – 2.02) |  |  |
|  |  | TT | 9/4 | | 2.03 (0.62 – 6.65) |  |  |

**Table S2** Association of ethnicity, age, gender and tobacco smoking status with GCLC-129 C/T (rs41303970) gene promoter polymorphism and IHD

| Groups | Inheritance model | Genotypes | Case/Control, n | | OR (95% CI) | χ2 | p-value |
| --- | --- | --- | --- | --- | --- | --- | --- |
| GCLC -129 C/T | | | | | | | |
| Ethnicity | | | | | | | |
| Kazakh | Dominant | CC+CT | 221/235 | 0.21 (0.04 – 0.98) | | 4.781 | 0.03 |
|  |  | TT | 9/2 | 4.79 (1.02 – 22.39) | |  |  |
|  | Recessive | CC | 168/191 | 0.65 (0.42 – 1.01) | | 3.740 | 0.05 |
|  |  | CT+TT | 62/46 | 1.53 (0.99 – 2.37) | |  |  |
| Russian | Dominant | CC+CT | 101/70 | 1.44 (0.09 – 23.46) | | 0.067 | 0.8 |
|  |  | TT | 1/1 | 0.69 (0.04 – 11.27) | |  |  |
|  | Recessive | CC | 66/49 | 0.82 (0.43 – 1.57) | | 0.349 | 0.6 |
|  |  | CT+TT | 36/22 | 1.21 (0.64 – 2.32) | |  |  |
| Age | | | | | | | |
| ˂45 | General | CC | 19/70 | | 0.50 (0.16 – 1.52) | 1.548 | 0.5 |
|  |  | CT | 5/9 | | 2.00 (0.60 – 6.64) |  |  |
|  |  | TT | 1/2 | | 1.65 (0.14 – 8.95) |  |  |
| >45 | General | CC | 238/198 | | 0. 77 (0.53 – 1.11) | 5.597 | 0.06 |
|  |  | CT | 88/61 | | 1.16 (0.80 – 1.69) |  |  |
|  |  | TT | 9/1 | | **7.15** (0.90 – 6.80) |  |  |
| Gender | | | | | | | |
| Males | Recessive | CC | 62/77 | | 0.91 (0.48 – 1.71) | 0.093 | 0.76 |
|  |  | CT+TT | 28/32 | | 1.10 (0.58 – 2.09) |  |  |
| Females | General | CC | 191/186 | | 0.60 (0.39 – 0.91) | 6.376 | 0.04 |
|  |  | CT | 71/43 | | 1.57 (1.02 – 2.41) |  |  |
|  |  | TT | 8/3 | | 2.33 (0.61 – 8.89) |  |  |
| Smoking | | | | | | | |
| Yes | Recessive | CC | 28/45 | | 0.62 (0.25 – 1.58) | 1.011 | 0.3 |
|  |  | CT+TT | 12/12 | | 1.61 (0.63 – 4.07) |  |  |
| No | General | CC | 229/223 | | 0.69 (0.47 – 1.00) | 6.748 | 0.03 |
|  |  | CT | 81/59 | | 1.29 (0.88 – 1.89) |  |  |
|  |  | TT | 10/2 | | 4.55 (0.99 – 0.94) |  |  |
